# The Catalytic Product of Pentachlorophenol 4-Monooxygenase is Tetra-chlorohydroquinone rather than Tetrachlorobenzoquinone

**DOI:** 10.2174/1874285800802010100

**Published:** 2008-08-12

**Authors:** Yunyou Su, Lifeng Chen, Brian Bandy, Jian Yang

**Affiliations:** College of Pharmacy and Nutrition, University of Saskatchewan, 110 Science Place, Saskatoon, Saskatchewan, Canada S7N 5C9, Canada

## Abstract

Pentachlorophenol 4-monooxygenase (PcpB) catalyzes the hydroxylation of pentachlorophenol in the pentachlorophenol biodegradation pathway in *Sphingobium chlorophenolicum*. Previous studies from two different research groups proposed oppositely that the catalytic product of PcpB was tetrachlorohydroquinone (TCHQ) and tetrachlorobenzoquinone (TCBQ). We re-examined the identity of the catalytic product of PcpB, because TCHQ and TCBQ are present in a redox-equilibrium in aqueous solutions and the chemical reagents NADPH, ethyl acetate and glutathione used for the product detection in the previous studies may shift the redox-equilibrium. In this study, we investigated the effects of NADPH, ethyl acetate and glutathione on the redox-equilibrium and product distribution. Under newly designed experimental conditions, we confirmed unambiguously that the catalytic product of PcpB is TCHQ instead of TCBQ. We also propose that TCBQ may be produced non-specifically by peroxidases within the bacterial cells and that TCBQ reductase (PcpD) might act as a self-protective rather than a PCP-degradation enzyme.

## INTRODUCTION

Pentachlorophenol (PCP), a chloroaromatic pesticide widely used to preserve lumber in the last century, is currently listed as one of the major environmental pollutants in North America [1-4]. In spite of being designed to resist biodegradation, PCP can be degraded by a number of soil and aquatic microorganisms such as *S. chlorophenolicum* [5-17]. The PCP biodegradation pathway in *S. chlorophenolicum*, which is one of the most characterized PCP biodegradation systems, converts PCP to 3-oxoadipate (3-OXO) using five catalytic enzymes [6-17].

**Fig. (1):**
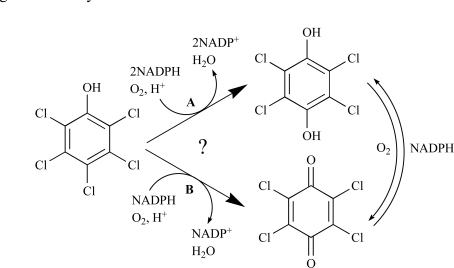
The proposed catalytic reaction of PcpB. **(A)**. Reported by Xun *et al.* [6,7,12,16]. **(B)**. Reported by Dai *et al.* [11].

The biodegradation of PCP in *S. chlorophenolicum* is highly inefficient mainly due to the low catalytic activity of pentachlorophenol 4-monooxygenase (PcpB), which is the first and rate-limiting enzyme in the pathway [6,7,11,12,16]. PcpB is a flavin-containing monooxygenase and catalyzes the hydroxylation of PCP using nicotinamide adenine dinucleotide phosphate (NADPH) as a co-substrate [6,7,11,12,16]. Previous studies by Xun *et al.* [6,7,12,16] identified tetrachlorohydroquinone (TCHQ) as the catalytic product of PcpB (Fig. **1**). Later Dai *et al.* [11] reported that the catalytic product of PcpB was tetrachlorobenzoquinone (TCBQ) rather than TCHQ (Fig. **1**) and that the reduction of TCBQ into TCHQ was catalyzed by a new enzyme, tetrachlorobenzoquinone reductase (PcpD), which was encoded by gene *pcpD*. Although TCBQ is now commonly accepted as the catalytic product of PcpB, we have a concern about the identity of the catalytic product of PcpB because the experiments undertaken by neither research group were sufficient to support their conclusion.

TCHQ and TCBQ are present in a redox-equilibrium in aqueous solutions except under rigorous anaerobic conditions. The redox-equilibrium can be easily shifted by reducing agents, oxidizing agents, and any other agents reactive with either TCHQ or TCBQ. Excessive NADPH used by Xun *et al.* [16] would non-enzymatically reduce TCBQ into TCHQ [18] if it were the catalytic product of PcpB, leaving only TCHQ detectable regardless the catalytic product of PcpB was TCHQ or TCBQ. On the other hand, ethyl acetate extraction and product trapping with glutathione (GSH) used by Dai *et al.* [11] were equally questionable. Contrary to hydroquinones, benzoquinones are usually poorly soluble in aqueous solutions [19]. Thus the extraction of the aqueous solution with ethyl acetate would favor oxidation of TCHQ, a hydroquinone, to TCBQ, a benzoquinone. As for the product trapping, glutathione (GSH) is a nucleophilic compound and should react quickly with the electrophilic TCBQ. Therefore, both ethyl acetate and GSH can shift the redox-equilibrium towards the TCHQ side. In summary, it is practically impossible to distinguish TCHQ from TCBQ unambiguously under the experimental conditions adopted by either research group.

In order to confirm the identity of the catalytic product of PcpB, we first studied the effects of NADPH, ethyl acetate and GSH on the redox-equilibrium between TCHQ and TCBQ. Based on the study results, we re-designed the experimental conditions for the hydroxylation reaction catalyzed by PcpB. TCHQ rather than TCBQ was confirmed to be the catalytic product of PcpB. In addition, we showed that peroxidases, which did not require PCP induction and were constitutively expressed within *S. chlorophenolicum* strain ATCC 39723, might be involved in the production of TCBQ from both PCP and TCHQ.

## MATERIALS AND METHODOLOGY

### Bacterial Culture Conditions

*S. chlorophenolicum* strain ATCC 39723, purchased from American Type Culture Collection (Manassas, USA), was cultured in mineral media (K2HPO4 0.65 g, KH2PO4 0.19 g, MgSO4 0.1 g, NaCl 2.0 g, glutamic acid 4.0 g, and FeSO4 3 mg). After inoculation with 1 mL of freshly grown cells, the bacterium was grown in 1 L of mineral media at 30 (C for 4 d before any experiment was undertaken.

### Expression and Purification of Recombinant His_6_-tagged PcpB

The *Escherichia coli* M15 cells over-expressing recombinant His_6_-PcpB were kindly provided by Dr. Shelley D. Copley of the University of Colorado at Boulder. The expression of PcpB was induced by isopropyl β-D-1-thiogalacto-pyranoside (IPTG) with a final concentration of 100 μM. After induction, the cells were grown for another 4 h before being harvested by centrifugation at 5,000 g for 20 min. The cell pellets obtained from 1 L cell cultures were stored at -80 (C. The purification of PcpB was performed at 4 ºC using affinity chromatography. Briefly, a frozen pellet from 1 L of cell culture was suspended in 50 mL of the lysis buffer (50 mM Tris-HCl, pH 7.7, 0.25 mM phenylmethanesulfonyl fluoride (PMSF), 1 μM pepstatin A, and 40 mg of lysozyme) and incubated with gentle rotation for 30 min. The cells were then subjected to further disruption by sonication using a Sonifier™ 150 sonicator. The cell lysate was centrifuged at 20,000 g for 30 min. The supernatant was mixed with 5 mL of Ni-NTA agarose media and shaken for 2 h before being packed into a column and washed thoroughly with the washing buffer (50 mM phosphate buffer, pH 7.7, 50 mM imidazole, 0.3 M NaCl, 0.25 mM PMSF, and 1 μM pepstatin A) at a flow rate of 2 mL/min until the elute UV absorbance was approximately zero. PcpB was eluted with 30 mL of the elution buffer (50 mM phosphate buffer, pH 7.7, 0.3 M NaCl, 250 mM imidazole, 0.25 mM PMSF, and 1 μM pepstatin A). The purity of PcpB in the elution fractions was examined on a 12% SDS-PAGE gel. Fractions containing pure PcpB were combined, buffer-exchanged to storing buffer (20 mM phosphate buffer, pH 7.0, 0.25 mM PMSF, 5% glycerol), concentrated to 15 mg/mL, and stored at -80(C.

### Calibration Curves for PCP, TCHQ and TCBQ

All experiments involved in the current study, including the standard curve calibrations, were undertaken in duplicate. PCP, TCHQ and TCHQ with higher than 98% purity were purchased from Sigma-Aldrich Canada (Oakville, Canada). To calibrate the standard curve for each compound, sample solutions with concentrations at 200 μM, 100 μM, 50 μM, and 25 μM were prepared by serial dilutions with deionized distilled water. The samples were then analyzed by HPLC on an Alltech C8 column. 30 μL of each sample was injected into the column and eluted with the mobile phase solvent of 60% acetonitrile and 40% acetic acid (0.1%) at 1 mL/min. The retention times for TCHQ, TCBQ and PCP were identified to be 7 min, 9.5 min, and 17.5 min, respectively. The area of the absorption peak for each sample on the HPLC histogram was calculated. A standard curve of each compound was obtained by a linear fit between the sample concentration and peak area. Quantification of PCP, TCHQ or TCBQ in other experimental samples was carried out by comparing their peak areas on the HPLC histogram against the respective standard curve.

### Reduction of TCBQ by NADPH

NADPH was added into 100 μL of 100 μM TCBQ water solutions to final concentrations of 0 μM, 50 μM, 100 μM, 250 μM, and 500 μM. The solutions were then allowed to sit at room temperature (~ 23 ºC) from 1 to 5 min before immediate loading on the Alltech C8 column for HPLC analysis. Being aware of that no chemicals would stop the reduction of TCBQ by NADPH without interfering with the redox-equilibrium, the reaction samples were analyzed by HPLC one at a time.

### Effects of Ethyl Acetate on the Redox-Equilibrium

1-mL solutions containing 100 μM TCHQ, 100 μM TCBQ, and 100 μM of both TCHQ and TCBQ were prepared in 50 mM phosphate buffer (pH 7.0). The UV-visible absorption spectra of the solutions were recorded on an Agilent 8453 diode array spectrophotometer (Agilent Technologies, Mississauga, Canada) at 25 (C using a 1-mL quartz cuvette of 10 mm path length. The UV-visible absorption spectra were baseline-corrected with the phosphate buffer. The solutions were then extracted with 1 mL ethyl acetate. The UV-visible absorption spectra of the extractions were also recorded with the baseline corrected with ethyl acetate.

### Effects of GSH on the Redox-Equilibrium

GSH was added into 100 μL solution of 100 μM TCHQ in 50 mM phosphate buffer, pH 7.0, with final concentrations of 0 μM, 50 μM, 100 μM, 200 μM, and 500 μM. The solutions were incubated at room temperature (~ 23 ºC) from 1 to 5 min before immediate loading on the Alltech C8 column for HPLC analysis. As with the reduction of TCBQ by NADPH, the reaction samples in this study were analyzed by HPLC one at a time.

### Measurement of Oxygen Consumption Rate

The oxygen consumption rate was determined in duplicate in a 1-mL enclosed system using a Hansatech oxytherm electrode unit (Hansatech Instruments, Norfolk, England) at 23.5 ºC. First, 1 mL of 50 mM phosphate buffer, pH 7.0, saturated with oxygen was placed into the electrode unit and the chamber was closed with the syringe-port plunger. Then, GSH was added to the unit with final concentrations of 0 μM, 50 μM, 100 μM, 200 μM, 500 μM, 1 mM, or 2 mM. Finally, TCHQ was injected into the unit with a final concentration of 100 μM. The oxygen consumption rate was measured after each injection.

### Detection of the Catalytic Product of PcpB

A 100 μL reaction solution containing 100 μM of PcpB and NADPH, respectively, was incubated at room temperature for at least 1 min prior to the addition of PCP to initiate the catalytic reaction of PcpB with a final concentration of 200 μM. The reaction was allowed to proceed for 1 min before being stopped either by quenching with1 M HCl or acetonitrile containing 0.1 M HCl or by quick centrifugation at 5,000 rpm for 30 seconds with an Amicon ultra-4 10 kDa filtration unit. The quenched samples were quickly centrifuged at 13,000 rpm for 30 seconds on a VWR Galaxy™ 14D microcentrifuge. 30 μL of the supernatant from the quenched samples or the filtrate from the Amicon centrifugation samples was immediately injected into the Alltech C8 column for HPLC analysis by eluting with the mobile phase solvent (60% acetonitrile and 40% acetic acid 0.1%) at 1 mL/min.

### Structural Model of PcpB

The crystal structure of the aromatic hydroxylase from *Streptomyces* strain pga64 (PDB ID: 2qa1) [20] was identified as the template to build the homology model of PcpB from the protein-protein BLAST (blastp) search against the Protein Data Bank using the amino acid sequence of PcpB. Pair-wise comparative sequence alignment between PcpB and the aromatic hydroxylase was done by ClustalW [21]. Ten PcpD initial models were built using software MODELLER V8.1 [22] with default parameters. The geometry and energy criteria for each initial model were evaluated by the programs PROCHECK [23], ProSa II [24] and Verify3D [25]. The model with best geometry and lowest energy was selected as the final structural model of PcpB. Energy minimization was not applied to the final model.

### Peroxidase Activity Assay

The peroxidase activity was assayed using the peroxidase/catechol color reaction method. Briefly, 1.5 mL of fresh cell culture of *S. chlorophenolicum* strain ATCC 39723 or 300 μL of the supernatant from the lysate of 5 mL cell culture was added into a 1.5-mL reaction mixture containing 1% pyrocatechol and 1.5% H_2_O_2_. The reaction was allowed to proceed for 5 min at room temperature (~ 23 ºC) before the color reaction was recorded.

## RESULTS

### NADPH Rapidly Reduces TCBQ to TCHQ

The non-enzymatic reduction of TCBQ by NADPH was studied at room temperature under four different molar ratios of NADPH and TCBQ. Both TCHQ and TCBQ were detected by high performance liquid chromatography (HPLC). Because either phosphate buffer (pH 7.0) or Tris-HCl buffer (pH 7.0) interfered with the detection of TCHQ by generating a strong solvent-front peak, the reduction reaction was undertaken in de-ionized distilled water (pH 6.8). As shown in Fig. (**2**), the reduction of TCBQ by NADPH was rapid at all four molar ratios. Reestablishment of the redox-equili-brium was achieved in less than 1 min. When the molar ratio was 0.5 or 1, approximately 50-65% of TCBQ was reduced to TCHQ. When the molar ratio was 2.5 or higher, almost all TCBQ was reduced to TCHQ. Therefore, in order to unambiguously confirm the identity of the catalytic product of PcpB, the molar ratio between NADPH and the product should always be less than 1 in the reaction system.

### Ethyl Acetate Extraction Increased the Oxidation of TCHQ

Because ethyl acetate extraction may have influenced previous results [11], we investigated the effect of ethyl acetate extraction on the redox-equilibrium between TCHQ and TCBQ using UV-visible spectroscopy. As shown in Fig. (**3**), the solution containing 100 μM TCHQ in 50 mM phosphate buffer exhibited a bell-shaped absorption peak centered at 304 nm; whereas the bell-shaped absorption peak for the 100 μM TCBQ solution was centered at 292 nm. The solution containing 100 μM of both TCHQ and TCBQ showed a much larger peak centered at 300 nm, which could be obviously viewed as a summation of the respective absorption peaks for TCHQ and TCBQ. The ethyl acetate extract of the 100 μM TCBQ solution gave a narrower absorption peak centered at 292 nm (Fig. **3**). However, the ethyl acetate extract of the 100 μM TCHQ solution gave two absorption peaks with maxima at 292 and 304 nm; and the ethyl acetate extract of the solution containing 100 μM TCHQ and 100 μM TCBQ showed a stronger absorption at 292 nm than the extract from 100 μM TCBQ solution and a weaker absorption at 304 nm than the extract from 100 μM TCHQ solution (Fig. **3**). These observations strongly suggested that TCHQ had been oxidized during the ethyl acetate extraction, and the extent of oxidation could approach 100%.

### GSH Shifted the Redox-Equilibrium Towards TCBQ

As a strong nucleophile, GSH may react rapidly with any electrophilic TCBQ formed through TCHQ oxidation to form GS-TriCBQ, dragging the redox-equilibrium towards TCBQ. Thus, we quantified the effects of GSH on the redox-equilibrium by adding GSH into 100 μM TCHQ water solution at five different concentrations using an HPLC method (Fig. **4**). GSH was indeed capable of shifting the redox-equilibrium to the TCBQ side and re-establishment of the redox-equilibrium occurred within 1 min. At 50 μM GSH, as much as 90% of TCHQ was converted to TCBQ, GS-TriCBQ or tetrachlorobenzosemiquinone. As GSH concentration was 100 μM or higher, only about 10-30% of TCHQ was converted. This is likely due to that the reaction system was in a stronger reducing environment and the oxidation of TCHQ to TCBQ was decreased. In order to confirm this rationale, we measured the oxygen consumption rate in a 1-mL enclosed system containing GSH and TCHQ in 50 mM phosphate buffer, pH 7.0. Addition of GSH into the phosphate buffer solution produced very little oxygen consumption (data not shown). However, subsequent addition of TCHQ into the phosphate buffer solution increased the oxygen consumption rate dramatically, ranging from 3.0 μM/min at 2 mM GSH to 9.5 μM/min at 200 μM GSH (Table **1**). It is obvious that the oxidation of TCHQ was much slower at higher GSH concentrations. This observation is consistent with the above HPLC analysis. Therefore, any nucleophilic compound such as GSH, which can shift the redox-equilibrium towards TCBQ, should be avoided in the characterization of PcpB.

### TCHQ Rather Than TCBQ was the Catalytic Product of PcpB

Contrary to the claim by Dai *et al.* [11] that TCHQ and TCBQ were usually co-eluted on HPLC, previous studies by Tjeerdema and Crosby [26] and our group showed that PCP, TCHQ and TCBQ could be easily separated by HPLC. Therefore, we decided to adapt the HPLC method to characterize the catalytic product of PcpB.

We first incubated 200 μL reaction mixture containing 100 μM PcpB and 100 μM NADPH at room temperature for at least 1 minute to ensure sufficient binding of NADPH to PcpB and reduction of the flavin. Unbound NADPH should therefore be present to a minimum amount in the reaction mixture. PCP, with the final concentration at 200 μM, was then added to the reaction mixture to initiate the catalytic reaction of PcpB for 1 min before the reaction was quenched with 1 M HCl. The quenched reaction mixture was immediately centrifuged at 13,000 rpm for 0.5 min and the supernatant was immediately loaded onto an Alltech C8 column for HPLC analysis. Under the current experimental conditions (equimolar enzyme:NADPH and excess PCP), NADPH would predominantly react with PCP, leaving the molar ratio between the un-reacted NADPH and the catalytic product of PcpB far below 1. Thus, the catalytic product of PcpB would be detected unambiguously no matter if it was TCHQ or TCBQ. As shown in Fig. (**5**), the HPLC histogram clearly showed the absorption peaks for TCHQ and PCP. But no trace of TCBQ was detected. In addition, the absorption peaks for both TCHQ and PCP were small, implying the small molecules bound significantly to the viscous gel of the denatured PcpB due to the large amount of enzyme used in the reaction and/or precipitated out due to lower solubility at acidic pH after quenching with HCl. In order to examine the possibility that TCBQ was the catalytic product of PcpB and completely precipitated out and/or bound to the denatured PcpB during the quenching, we repeated the above experiment by stopping the reaction either with 0.1 M HCl in acetonitrile as the quenching reagent to increase the presence of TCBQ in the supernatant or with the filtration method using an Amicon centrifugal filter unit with molecular weight cutoff of 10 kDa. However, we still could not detect any TCBQ in the reaction mixture. We observed that the absorption peak for PCP in the Amicon filtrate was still small, suggesting that PCP bound specifically and/or non-specifically to PcpB because of the large amount of enzyme used. In addition, that the absorption peak for TCHQ in the filtrate was also small suggests that PcpB, its catalytic product, and flavin (either FMN or FAD) were likely present as a ternary complex, in which TCHQ could be trapped much easier by GSH to form GS-TriCBQ.

To further investigate the catalytic mechanism, a structural model of PcpB was constructed by homology modeling using the coordinates of the aromatic hydroxylase from *Streptomyces* strain pga64 (PDB ID: 2qa1) as the template [20]. The active site of PcpB, as defined by Nakamura *et al.* [27], was highly positively charged (Fig. **6**). The positive electrostatic potentials make it favorable for PcpB to produce TCHQ instead of TCBQ, because the electrophilic TCBQ is not stable in a positively charged environment and the reaction catalyzed by PcpB would have to cross a much higher energy barrier if the catalytic product were TCBQ.

## DISCUSSION

**Fig. (2):**
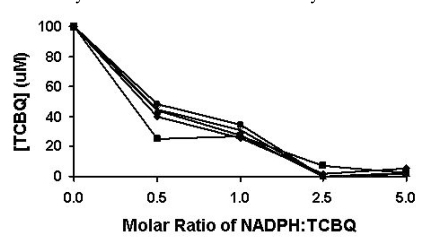
Reduction of TCBQ by NADPH under different molar ratios be-tween NADPH and TCBQ. The reduction reaction was monitored from 1 to 5 min. The results at the 1, 2, 3, 4, and 5 min were represented by solid square, solid diamond, solid triangle, asterisk, and solid circle, respectively.

In the current studies, we showed that reducing agent NADPH rapidly reduced TCBQ to TCHQ (Fig. **2**). To confirm the identity of the catalytic product of PcpB, the experiment should be designed carefully to ensure that the unreacted NADPH was less than the catalytic product in the reaction system. Although Xun *et al.* [7] used the substrates NADPH and PCP at 1:1 ratio in their initial characterization of PcpB, the low catalytic activity of PcpB resulted in the molar ratio between NADPH and the catalytic product of PcpB being much higher than 1 at any time during the reaction. The inefficiency of PcpB led Xun *et al.* to use a much higher molar ratio (16,000) between NADPH and PCP in their subsequent studies on PcpB [16]. However, under either circumstance, only TCHQ would be identified due to the high molar ratio between NADPH and the product of PcpB regardless of whether the initial product was TCHQ or TCBQ.

**Fig. (3):**
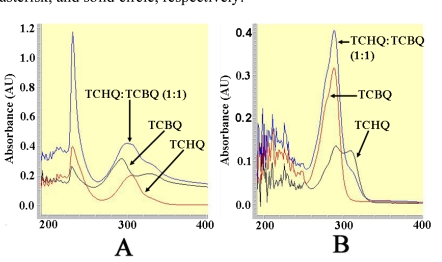
The UV-visible spectra for the 1-mL solutions containing 100 µM TCHQ, 100 µM TCBQ, and 100 µM of both TCHQ and TCBQ, respectively, in either 50 µM phosphate buffer, pH 7.0 **(A)** or ethyl acetate extract **(B)**.

**Fig. (4):**
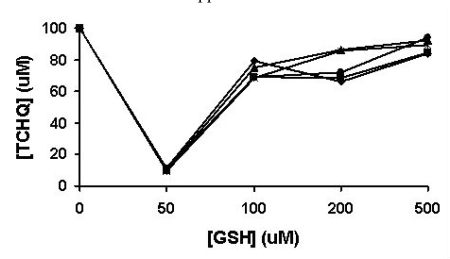
The effects of GSH on the redox-equilibrium between TCHQ and TCBQ. The reaction was monitored from 1 to 5 min. The results at the 1, 2, 3, 4, and 5 min were shown in solid square, solid diamond, solid triangle, asterisk, and solid circle, respectively..

**Fig. (6):**
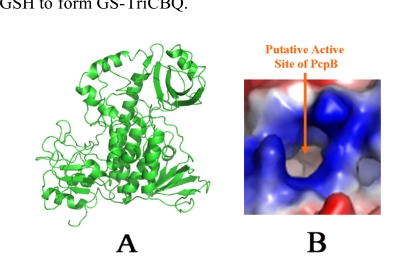
Homology structural model of PcpB. **(A).** A ribbon representation. **(B).** Electrostatic potential distribution at the proposed active site by Nakamura *et al.* [27].

Dai *et al.* provided two key pieces of evidence to support TCBQ as the catalytic product of PcpB [11]. The first piece of evidence was the identification of TCBQ by gas chromatography-mass spectrometry (GC/MS) from the ethyl acetate extract of the PcpB reaction mixture. The ethyl acetate extraction step was reported to be necessary in the GC/MS detection [11]. As described in the introduction section, benzoquinones are less soluble than hydroquinones in aqueous solutions [19] and organic solvent extraction may increase the oxidation of hydroquinones to the corresponding benzoquinones. In the current studies we showed that ethyl acetate extraction increased the oxidation of TCHQ to TCBQ (Fig. **3**). Therefore, the ethyl acetate extraction experiment undertaken by Dai *et al.* [11] was not sufficient to support TCBQ as the catalytic product of PcpB. The second piece of evidence was the formation of 2,3,5-trichloro-6-*S*-glutathionyl-benzoquinone (GS-TriCBQ) upon adding GSH to the PcpB reaction mixture [11]. However, the current studies also showed that GSH shifted the redox equilibrium towards TCBQ and the oxidation of TCHQ was slower at higher GSH concentrations (Fig. **4** and Table **1**). Dai *et al.* reported a 95% conversion rate for PCP by using 2 mM GSH to trap the catalytic product of PcpB [11]. This conversion rate would be much higher than our observation reported above if TCHQ were the catalytic product of PcpB. But we could not conclude TCBQ as the catalytic product of PcpB either, because the high conversion rate could have been obtained with TCHQ as the catalytic product of PcpB for the following reasons. First, the reaction carried out by Dai *et al.* was at 37 ºC; whereas ours was only at room temperature (~ 23 ºC). The oxidation of TCHQ to TCBQ would be more than twice as fast at 37 ºC. Secondly, the reaction undertaken by Dai *et al.* was at pH 8.0; whereas our experiment was done at pH 6.8. TCHQ would be oxidized much faster in alkaline solutions than in acidic solutions [28]. Finally, the usage of a large amount of PcpB in the reaction by Dai *et al.* might result in the formation of the transient state ternary complex of PcpB, the catalytic product, and flavin. GSH could directly react with the ternary complex to form GS-TriCBQ, which would be much faster than first shifting the redox-equilibrium towards the TCBQ side and then reacting with the TCBQ. Thus, trapping the catalytic product of PcpB by GSH is unlikely to be an appropriate method to detect the identity of the catalytic product of PcpB.

Under newly designed experimental conditions, we showed unambiguously that TCHQ rather than TCBQ was the catalytic product of PcpB. Then, two important questions that are needed to be addressed are what role PcpD plays and how TCBQ is produced during the biodegradation of PCP, since previous studies by both Dai *et al. *and our group confirmed that PcpD was indeed a TCBQ reductase instead of a monooxygenase reductase in spite of its low but statistically significant activity [11,29]. To answer these two questions, we decided to analyze the published study results on the *pcpD*-knockout strain of *S. chlorophenolicum *ATCC 39723 [11].

The *pcpD*-knockout strain of ATCC 39723 was able to degrade PCP at lower (100 μM) but not higher (300 μM) concentrations [11]. Three explanations were provided by Dai *et al.* for this phenomenon [11]. The first explanation was that the absence of PcpD resulted in accumulation of the toxic TCBQ that overcame the bacterial cells at higher concentrations of PCP (such as 300 μM). If the explanation were valid, we would expect a deceleration of the degrading rate even at lower concentrations of PCP (such as 100 μM) because PcpB was inhibited by TCBQ in a concentration-dependent manner in the range of 0-80 μM and lost almost all of its catalytic activity at 80 μM of TCBQ [11]. However, the PCP degrading rate of the *pcpD*-knockout strain was about the same throughout the whole degradation process in the presence of 100 μM of PCP [11], indicating that TCBQ was not accumulating within the bacterial cells. The second explanation was that PcpD might be involved in the bacterial response towards the toxicity of PCP or its metabolites, and the third explanation was that the knockout of gene *pcpD* might alter the expression level of the regulatory gene *pcpR*. But currently there are no experimental evidences available to support these two explanations.

**Fig. (7):**
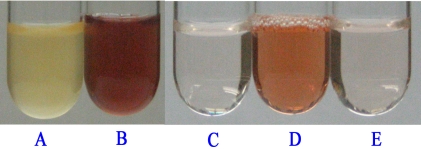
Peroxidase activity assay of the *S. chlorophenolicum* strain ATCC 39723 cell culture and its cell lysate supernatant. **(A)**. 1.5 mL bacterial cell culture + 1.5 mL reaction mixture containing 1% pyrocatechol; **(B)**. 1.5 mL bacterial cell culture + 1.5 mL reaction mixture containing 1% pyrocatechol and 1.5% H_2_O_2_; **(C)**. 300 µL of 50 mM phosphate buffer, pH 7.0 + 1.5 mL reaction mixture containing 1% pyrocatechol and 1.5% H_2_O_2_; **(D)**. 300 µL of cell lysate supernatant + 1.5 mL reaction mixture containing 1% pyrocatechol and 1.5%  H_2_O_2_.; and **(E)**. 300 µL of boiled cell lysate supernatant + 1.5 mL reaction mixture containing 1% pyrocatechol and 1.5% H_2_O_2_.

Compared to the wild-type, the *pcpD*-knockout strain showed a much slower degradation rate at 100 μM of PCP [11], implying that there was a small amount of TCBQ in the bacterial cells. However, the almost unchanged PCP degradation rate suggested that the *in* *vivo* concentration of TCBQ was constant. It was likely that TCBQ was produced by other enzymes rather than PcpB and/or by non-enzymatic oxidation of TCHQ, because TCBQ would accumulate to higher concentrations, inhibit the rate-limiting enzyme PcpB, and decrease the PCP degradation rate if it were produced by PcpB. Taking into consideration that PCP could be converted to TCBQ *in vivo* by peroxidases in fungi [30] and *in vitro* by horseradish peroxidase [31,32], we suspected that TCBQ was also produced from PCP by at least one peroxidase in *S. chlorophenolicum* strain ATCC 39723. We thus measured the peroxidase activity of the cell culture of strain ATCC 39723 and the supernatant of the bacterial cell lysate (Fig. **7**). Peroxidases were observed to be consistently expressed and did not require PCP induction in the strain ATCC 39723, implying that the conversion of PCP to TCBQ by peroxidases was non-specific. The peroxidases might also non-specifically oxidize TCHQ, the catalytic product of PcpB, to TCBQ. Based on the above observation, we hypothesized that PcpD acted as a protective enzyme against the cyto-toxicity caused by TCBQ rather than as a PCP-degradation enzyme, because TCBQ has been shown to be extremely toxic to *E. coli* spheroplasts at concentrations lower than 1 μM [9]. Our current hypothesis can readily explain the experimental results on the *pcpD*-knockout strain of ATCC 39723. At lower concentrations of PCP (such as 100 μM), only a small amount of TCBQ was produced non-specifically by the peroxidases, causing a partial inhibition of PcpB but not high toxicity to the bacterial cells. As PCP was increased to higher concentrations (such as 300 μM), more TCBQ was produced by the peroxidases, leading to severe toxicity to the bacterial cells and complete inhibition of the bacterial growth. Further studies, including identifying and cloning the peroxidases, are required to fully understand the PCP biodegradation pathway and the cyto-protection mechanism in *S. chlorophenolicum*.

## CONCLUSION

In the current study, we showed that chemical reagents NADPH, ethyl acetate and GSH were capable of shifting the redox-equilibrium between TCHQ and TCBQ. Under newly designed experimental conditions, TCHQ rather than TCBQ was identified unambiguously to be the catalytic product of PcpB. Re-analysis of the study results on the *pcpD*-knockout strain of *S.* *chlorophenolicum* ATCC 39723 implicated PcpD as a protective enzyme against the cyto-toxicity of TCBQ rather than as a PCP-degradation enzyme.

## Figures and Tables

**Fig. (5):**
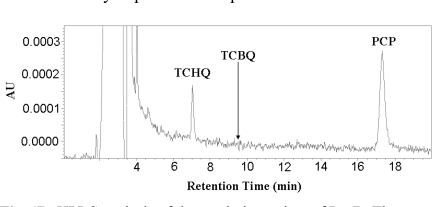
HPLC analysis of the catalytic product of PcpB. The retention time for TCBQ (9.5 min) was indicated by an arrow in the histogram.

**Table 1.** Oxygen Consumption Rate (Average ± Standard Deviation of two Independent Measurements) from 100 µ M TCHQ Incubated in an Enclosed Reaction System with Different GSH Concentrations at 23.5 °C

GSH (mM)	Oxygen Consumption (mM/min)
0	5.4 ± 0.3
50	9.1 ± 0.1
100	8.1 ± 0.4
200	9.5 ± 0.3
500	6.3 ± 0.3
1000	4.2 ± 0.5
2000	3.0 ± 0.3
